# Designing Ultraflexible Perovskite X‐Ray Detectors through Interface Engineering

**DOI:** 10.1002/advs.202002586

**Published:** 2020-11-13

**Authors:** Stepan Demchyshyn, Matteo Verdi, Laura Basiricò, Andrea Ciavatti, Bekele Hailegnaw, Daniela Cavalcoli, Markus Clark Scharber, Niyazi Serdar Sariciftci, Martin Kaltenbrunner, Beatrice Fraboni

**Affiliations:** ^1^ Division of Soft Matter Physics Institute for Experimental Physics Johannes Kepler University Linz Altenberger Strasse 69 Linz 4040 Austria; ^2^ Soft Materials Lab Linz Institute of Technology Johannes Kepler University Linz Altenberger Strasse 69 Linz 4040 Austria; ^3^ Department of Physics and Astronomy University of Bologna Viale Berti Pichat 6/2 Bologna 40127 Italy; ^4^ National Institute for Nuclear Physics – INFN section of Bologna Bologna Italy; ^5^ Linz Institute for Organic Solar Cells (LIOS) Institute of Physical Chemistry Johannes Kepler University Linz Altenberger Strasse 69 Linz 4040 Austria

**Keywords:** interface engineering, perovskites, ultraflexible, X‐ray detectors

## Abstract

X‐ray detectors play a pivotal role in development and advancement of humankind, from far‐reaching impact in medicine to furthering the ability to observe distant objects in outer space. While other electronics show the ability to adapt to flexible and lightweight formats, state‐of‐the‐art X‐ray detectors rely on materials requiring bulky and fragile configurations, severely limiting their applications. Lead halide perovskites is one of the most rapidly advancing novel materials with success in the field of semiconductor devices. Here, an ultraflexible, lightweight, and highly conformable passively operated thin film perovskite X‐ray detector with a sensitivity as high as 9.3 ± 0.5 µC Gy^−1^ cm^−2^ at 0 V and a remarkably low limit of detection of 0.58 ± 0.05 μGy s^−1^ is presented. Various electron and hole transporting layers accessing their individual impact on the detector performance are evaluated. Moreover, it is shown that this ultrathin form‐factor allows for fabrication of devices detecting X‐rays equivalently from front and back side.

## Graphical abstract

Ultraflexible, lightweight, and highly conformable thin film perovskite X‐ray detectors are presented here. An evaluation of the individual impact on the detector performance of various electron and hole transporting layers is provided. Such devices are passively operated and show a high sensitivity and a remarkably low limit of detection, allowing also for isotropic detection.

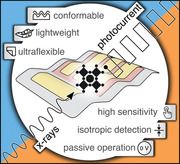

## 1 Introduction

The world around us consists of a myriad of objects with curved geometries and complex surfaces, yet the majority of sensors and detectors that we use to study them are rigid and planar. Confronting this incompatibility will allow for applications impossible with current technologies.

Ultraflexible, low‐cost, and highly sensitive high energy radiation detectors are of great interest to the fields of medical diagnostics, dosimetry, industrial inspection, security, and defence. Low weight and high conformability of X‐ray wearable dosimeters are appealing features for astronauts, nuclear power plants, and laboratory workers, as well as for imagers used in structural inspection and cultural heritage preservation.

State‐of‐the‐art solid state X‐ and gamma‐ray detectors for large area applications based on silicon (Si), amorphous selenium (a‐Se),^[^ ^1^ ^]^ mercury(II) iodide (HgI_2_), and cadmium zinc telluride (CdZnTe)^[^ ^2^ ^]^ are mechanically stiff, difficult to scale up, and have high operating voltage. Organic semiconductors were the first group of materials that was able to overcome these issues, offering liquid phase, low‐temperature, and low‐cost deposition techniques that are scalable to large flexible substrates. Mechanical flexibility, high X‐ray sensitivity (up to 1.3 × 10^4^ µC Gy^−1^ cm^−2^)^[^ ^3^ ^]^ and low limit of detection (down to 0.29 μGy s^−1^)^[^ ^4^ ^]^ have been demonstrated for both organic single crystal^[^ ^5^, ^6^, ^7^, ^8^ ^]^ and thin film direct X‐ray detectors.^[^ ^9^, ^10^, ^11^ ^]^ Despite these encouraging results, organic semiconductors are intrinsically low‐Z materials, therefore resulting in subpar high energy photon absorption. Blends of organic semiconductors and heavy inorganic nanoparticles or lead‐based quantum dots have been proposed to overcome such issues.^[^ ^11^, ^12^, ^13^, ^14^, ^15^, ^16^ ^]^ On one hand, this approach offers a strategy to improve the material attenuation fraction, but on the other hand, it is intrinsically limited by the maximum nanoparticle concentration that can be dispersed in the blend before clustering and agglomeration occurs, resulting in electronic transport degradation.

Recently, lead‐halide perovskites emerged as an auspicious novel materials family for X‐ and gamma‐ray detection.^[^ ^17^, ^18^, ^19^ ^]^ Their success can be attributed to strong absorption of ionizing radiation due to presence of heavy atoms (Pb, I, and Br), high charge carrier mobilities, long exciton diffusion, long charge carrier lifetime, and excellent optical properties.^[^ ^20^, ^21^, ^22^, ^23^, ^24^ ^]^ Single crystal^[^ ^19^, ^25^, ^26^, ^27^, ^28^, ^29^, ^30^, ^31^ ^]^ and thick film^[^ ^32^, ^33^, ^34^, ^35^, ^36^ ^]^ perovskite X‐ray detectors have been the focal point of current research, often incorporated in a lateral photoconductor radiation detector architecture. This kind of devices show an outstanding sensitivity as well as fast, stable, and reproducible response. Increasing the thickness of crystals or films has been the primary way to raise the total radiation absorbance and thus improve the detector efficiency. Nevertheless, limited attention has been devoted to thin film perovskite ionizing radiation detectors. Vertical configuration photodiode architecture based on thin films allows for lower dark current, faster response and stands out as the only viable candidate for flexible device implementation.^[^ ^37^, ^38^ ^]^

Flexible perovskite X‐ray detectors have been reported by Liu et al.,^[^ ^39^ ^]^ based on colloidal all‐inorganic CsPbBr_3_ perovskite quantum dots (QDs) printed over pre‐patterned electrodes onto PET (polyethylene terephthalate) substrates, with a photoconductor architecture. Despite the good X‐ray detection performance (sensitivity of 17.7 µC Gy^−1^ cm^−2^) at 0.1 V for an effective area of 0.06 mm^2^, the use of QDs suspensions and the photoconductor architecture pose problems for material stabilization, transport control, and detection performance.

However, these issues can be overcome through interface engineering using photodiode architecture^[^ ^40^, ^41^, ^42^ ^]^ that also greatly benefits from a vast knowledge base of highly performing rigid and ultraflexible perovskite solar cells.^[^ ^43^, ^44^ ^]^ X‐ray detectors fabricated on a flexible substrate with photodiode architecture were reported by Gill et al.,^[^ ^45^ ^]^ achieving detection performance of 0.2 µC Gy^−1^ cm^−2^ at 0 V, though not accessing device flexibility. Additionally, Mescher et al.^[^ ^46^ ^]^ also demonstrated flexible photodiode X‐ray detectors printed onto 25 µm polyethylene naphthalate foils, achieving maximum 59.9 µC Gy^−1^ cm^−2^ at 0.1 V and showing stable performance with bending radii down to 3 mm. A more detailed and complete comparison with the state‐of‐the‐art perovskite film‐based direct X‐ray detectors reported in literature in the last few years is provided by Table S1 (Supporting Information).

Here, we report the first ultraflexible, lightweight, and highly conformable X‐ray detectors based on mixed‐cations mixed‐halide perovskite composition with excellent performance that was achieved by means of a thorough interfacial engineering study. Five different interlayer configurations have been implemented. We employ either phenyl‐C_61_‐butyric acid methyl ester (PCBM) or *N*,*N*′‐dimethyl‐3,4,9,10‐perylentetracarboxylic diimide (PTCDI) as electron transport layer (ETL), and poly(3,4‐ethylenedioxythiophene):poly(styrenesulphonate) (PEDOT:PSS) or NiO*_x_* as hole transport layer (HTL). The here reported devices can be successfully operated at 0 V, i.e., passive mode operation. A sensitivity of 9.3 ± 0.5 µC Gy^−1^ cm^−2^ with an active area of 0.05 cm^2^ at 0 V and limit of detection down to 0.58 ± 0.05 μGy s^−1^ are achieved, both setting a current record for passive thin film perovskite X‐ray detectors.^[^ ^37^, ^38^, ^45^ ^]^ Moreover, these ultraflexible devices allow for isotropic operation, reliably detecting X‐rays impinging either on the back or the front side of the detector.

## 2 Results

### 2.1 Highly Conformable X‐Ray Detectors

**Figure 1:**
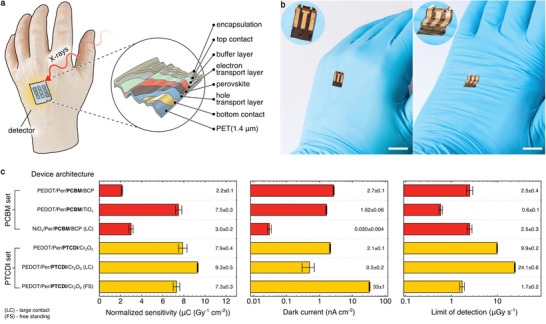
Ultraflexible X‐ray detectors. a) Schematic of the thin film perovskite X‐ray detector fabricated on 1.4 µm PET foil. b) Photograph of a X‐ray detector on a laboratory glove, highlighting its ability to conform to complex surfaces (scale bars 1 cm, insets magnified twice). c) Summary of the interface engineering study and individual device architecture influence on principal detector performance parameters (sensitivity at 0 V, dark current, and limit of detection) for phenyl‐C_61_‐butyric acid methyl ester (PCBM) (red) and*N*,*N*′‐dimethyl‐3,4,9,10‐perylentetracarboxylic diimide (PTCDI) based (yellow) set of devices. Each structure shows particular advantages over its alternatives, e.g., top sensitivity of 9.3 ± 0.5 µC Gy^−1^ cm^−2^in poly(3,4‐ethylenedioxythiophene)(PEDOT)/Per/PTCDI/Cr_2_O_3_(LC) [LC, large contact], lowest dark current of 0.030 ± 0.04 nA cm^−2^in NiO*_x_*/Per/PCB/BCP (LC), or best limit of detection of 0.58 ± 0.05 μGy s^−1^in PEDOT/Per/PCBM/TiO*_x_*structure.

Here, we introduce an ultraflexible X‐ray detector, fabricated using 500 nm mixed‐cation mixed‐halide perovskite composition (Cs_0.05_(FA_0.83_MA_0.17_)_0.95_PbI_3‐_ *_x_*Br*_x_*) as a high energy photon absorption layer, incorporated in an inverted (p‐i‐n) photodiode configuration on 1.4 µm thick PET foil substrate (**Figure** **1**a). The employment of a thin perovskite absorbing layer allows both efficient charge collection and scalability onto ultraflexible substrates. Ultraflexibility in electronics, referring to the design approach that allows repeated bending to radii considerably below 1 mm, has been explored through various means including overall thinning‐down of the device and using various ultrathin substrates.^[^ ^47^, ^48^, ^49^, ^50^, ^51^ ^]^ Indeed, ultrathin PET foil substrate (1.4 µm thick), commonly used in foil capacitors and other imperceptible electronics,^[^ ^44^, ^49^ ^]^ grants these devices extreme flexibility and conformability. Figure 1b demonstrates exceptional structural adaptability and pliancy of the detector to intricate and highly structured surfaces with small curvature radii, e.g., wrinkles on the surface of a laboratory safety glove.

We examine several ETL and HTL in order to investigate their contribution on principal detector performance parameters such as sensitivity, dark current, and limit of detection (Figure 1c and Table S2, Supporting Information). All the normalized sensitivity values reported in Figure 1c and throughout this work, have been calculated as the linear fits’ slope of the plots of the X‐ray induced photocurrent density as a function of the incident radiation dose rate. The first set of devices comprises detectors employing solution processed PCBM as ETL, augmented with either bathocuproine (BCP) or TiO*_x_* hole blocking buffer layer (Figure 1c, red). These devices use Al as the top metal contact and UV curable epoxy in combination with polypropylene (PP) foil as an encapsulation layer. The other set of detectors employs vacuum sublimed PTCDI as ETL with an additional Cr_2_O_3_ at the electrode interface (Figure 1c, yellow). Here, the Au top electrode is covered with ≈1 µm‐thin spin‐coated polyurethane (PU) layer serving as a mechanical protection layer. This results in an ultrathin architecture with about 3 µm total device thickness, therefore rendering it ultraflexible. Furthermore, the devices contain either PEDOT:PSS (formulation PH1000 Clevious) or solution processed NiO*_x_* as HTL. In order to improve charge collection, devices marked LC (Large Contact) have 100 nm‐thin Au bottom contact extending over the entire detector active area. Since transparent conductive electrodes are not required for X‐ray photodetector operation, thermally evaporated thin metal layer serves as readily deposited highly conductive X‐ray transparent bottom contact.

### 2.2 PCBM‐Based Devices

**Figure 2:**
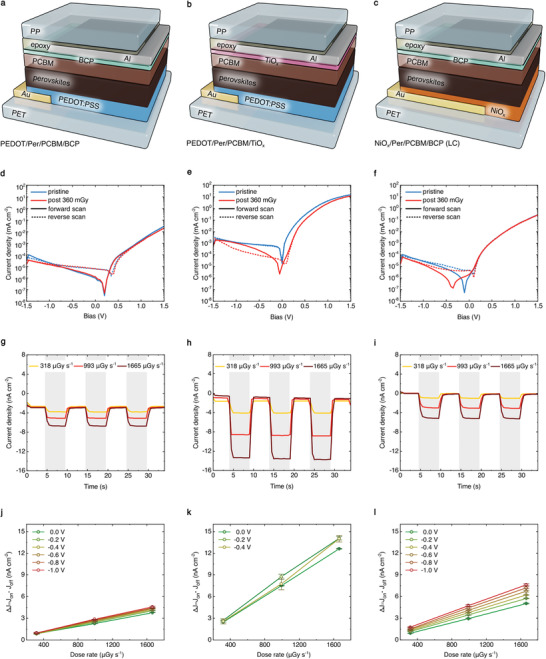
Phenyl‐C_61_‐butyric acid methyl ester (PCBM) based perovskite X‐ray detectors. Device architectures of perovskite high energy photon absorber interfaced with a) PEDOT:PSS (where PEDOT is poly(3,4‐ethylenedioxythiophene; PSS is poly(styrenesulphonate) and PCBM/BCP (where BCP is bathocuproine), b) PEDOT:PSS and PCBM/TiO*_x_*, and c) NiO_*x*_and PCBM/BCP using Al as the top contact and epoxy/polypropylene (PP) as encapsulation. d–f)*J*−*V*characteristics of the three structures acquired in dark conditions, before and after the X‐ray exposure, showing relatively low hysteresis of PCBM‐based devices. g–i) Dynamic detector response under X‐ray beam (40 keV) at different incident dose rate (318, 992, and 1885 μGy s^−1^). The gray boxes identify the time window when the X‐ray beam is turned on (5 s ON and 5 s OFF). Due to the low hysteresis, PCBM‐based detectors show well reproducible dark current. j–l) X‐ray induced photocurrent as a function of the incident radiation dose rate for different reverse biases, down to −1 V. PEDOT/Per/PCBM/TiO*_x_*architecture yields most sensitive device amongst PCBM‐based detectors.

PCBM is one of the standard ETL materials used in inverted perovskite solar cells and photodiodes due to its simple low‐temperature solution processing, compatibility with flexible form‐factor, and reduction of current hysteresis in the devices. The large density of charge carrier traps at the perovskite surface has been identified as the major reason for the strong hysteresis effect, which can be remedied with an introduction of a fullerene‐based material at the cathode interface. PCBM has been reported to passivate the perovskite surface by reducing interface charge recombination, resulting in reduced hysteresis and overall improved device performance.^[^ ^52^ ^]^ Here, we test several PCBM‐based X‐ray photodiode architectures containing BCP (**Figure** **2**a,c) or TiO*_x_* (Figure 2b) as an additional buffer layer, that serve the role of reducing the electron injection barrier formed at the PCBM/electrode interface.^[^ ^53^ ^]^ We observe very low hysteresis in *J−V* measurements carried out on pristine devices (Figure 2d–f). Decreased hysteresis directly translates to more reproducible dark current behavior, thus leading to more electrically stable X‐ray detectors. This is confirmed by measuring the dynamic response of the detector (Figure 2g–i) at 0 V under an irradiation with a 40 kVp X‐ray beam (spectrum reported in Figure S1, Supporting Information) at three different dose rates (318, 992, and 1885 μGy s^−1^), and over three ON/OFF beam cycles (5 s ON and 5 s OFF). We find that all PCBM‐based detector architectures show a box‐like response to X‐rays, stability over the three irradiation cycles, and a linear increase of the signal amplitude with increasing dose rate. In order to access the influence of the applied bias on device performance, the photocurrent is measured as a function of the dose rate at a voltage ranging between 0 and −1 V (Figure 2j–l). The X‐ray induced photocurrent density is calculated as $\Delta J=J_{\mathrm{on}}-J_{\mathrm{off}}$, where *J*_on_ is the detector current density during the X‐ray irradiation and *J*_off_ is the dark current density. Low bias dependence of photocurrent and sensitivity (see Figure S2, Supporting Information) suggests that the built‐in electric field of the p‐i‐n diode structure is sufficient to collect all the charges generated by the absorption of the X‐rays in the 500 nm thick perovskite layer. The possibility of operating these detectors in a passive mode is extremely relevant for wearable flexible electronic applications, for the reduction of user's risk and the assurance of sensing performance even in the case of interaction with the skin surface electric potential, and where ultraflexibility constitutes a huge added value in terms of lightweight and human comfort.

Further, we examine the influence of ETL/electrode buffer interlayers on the overall performance of X‐ray photodiodes by comparing the behavior of PEDOT/Per/PCBM/BCP (Figure 2a) and PEDOT/Per/PCBM/TiO*_x_* (Figure 2b) device architectures. We note that in our previous work^[^ ^38^ ^]^ we found a significant dependence of the photocurrent and sensitivity on the bias in X‐ray photodiodes with similar perovskite active layer thickness (450 nm). This could be ascribed to the employment of a much thicker ETL, i.e., mesoporous TiO*_x_* layer^[^ ^54^ ^]^ (about 300 nm instead of 5–10 nm for the buffer layers used in this work), which possibly gives a nonnegligible contribution to the X‐rays photogenerated charges (the X‐rays attenuated fraction of the different materials is reported in Figure S3, Supporting Information). In this study, detectors containing thin TiO*_x_* interlayer yield the highest X‐ray induced photocurrent and highest sensitivity of 7.5 ± 0.3 µC Gy^−1^ cm^−2^ at 0 V within PCBM‐based set of architectures (Figure 1c). The enhanced performance of these devices can be ascribed to TiO*_x_* high electron affinity (ETL characteristic) and lower conduction band (CB) level (CB(TiO*_x_*)  = −4.1 eV, LUMO(BCP)  = −3.5 eV, where LUMO stands for Lowest Unoccupied Molecular Orbital). This gives rise to lower characteristic charge transport resistance values and improved electron extraction, while still ensuring ohmic contact and reduced charge accumulation^[^ ^55^ ^]^ (see energy band diagrams in Figure S4, Supporting Information). It has also been suggested that TiO*_x_* plays a role in preventing perovskite halide ion diffusion through PCBM that can then react with the metal electrode, thus avoiding the formation of insulating layers at the PCBM/electrode interface.^[^ ^55^, ^56^ ^]^ Additionally, we observe lower dark current in PEDOT/Per/PCBM/TiO*_x_* devices when compared to PEDOT/Per/PCBM/BCP, therefore presenting another advantage of using TiO*_x_* as ETL/electrode buffer layer (Figure 1c). The major contributing factors to photodiode dark current are reverse saturation current and current resulting from parallel shunt pathways.^[^ ^57^ ^]^ Hailegnaw et al. reported that use of TiO*_x_* considerably reduces the surface roughness and significantly improves PCBM surface coverage, thus increasing the parallel shunt resistance and contributing significantly to dark current reduction.^[^ ^55^ ^]^ Therefore, the combination of high X‐ray induced photocurrent and low dark current in PEDOT/Per/PCBM/TiO*_x_* yields a low limit of detection of about 0.58 ± 0.05 μGy s^−1^ (defined as three times the detector noise) (Figure S5, Supporting Information). This value is the lowest among perovskite thin film detectors^[^ ^39^, ^46^, ^58^ ^]^ and it compares well with other thick perovskite X‐ray detectors,^[^ ^18^, ^34^ ^]^ while also being considerably below the medical requirements for diagnostics (≈5 μGy s^−1^).^[^ ^59^, ^60^ ^]^

We also compare the influence of HTL by examining the performance of PEDOT/Per/PCBM/BCP (Figure 2a) and NiO*_x_*/Per/PCBM/BCP (LC) (Figure 2c) detector architectures. While devices that use either NiO*_x_* or PEDOT:PSS HTL have comparable (that of NiO*_x_* is only slightly higher) sensitivities (Figure 1c and Figure S2, Supporting Information), NiO*_x_*/Per/PCBM/BCP detectors show exceptionally low dark current. We attribute this behavior to high CB level of NiO*_x_* and thus excellent electron blocking properties of this HTL. Nevertheless, the use of PEDOT allows for higher degree of flexibility of the device, thus posing an advantage over NiO_*x*_.

### 2.3 PTCDI‐Based Devices

PTCDI (Pigment Red 179) is a small molecule organic semiconductor that belongs to perylene diimide derivative family. Commonly used for industrial‐scale automotive and fiber coating, it is well known for its exceptional chemical, thermal, photo, and weather stability. In addition to environmental durability of PTCDI, its low‐lying HOMO level (−6.3 eV), high electron mobility (≈1–10 cm^2^ V^−1^ s^−1^), electron affinity, and lower rigidity when compared to inorganic materials like TiO*_x_*, makes this material an excellent nonfullerene alternative ETL for flexible perovskites and organic solar cells.^[^ ^61^ ^]^

**Figure 3:**
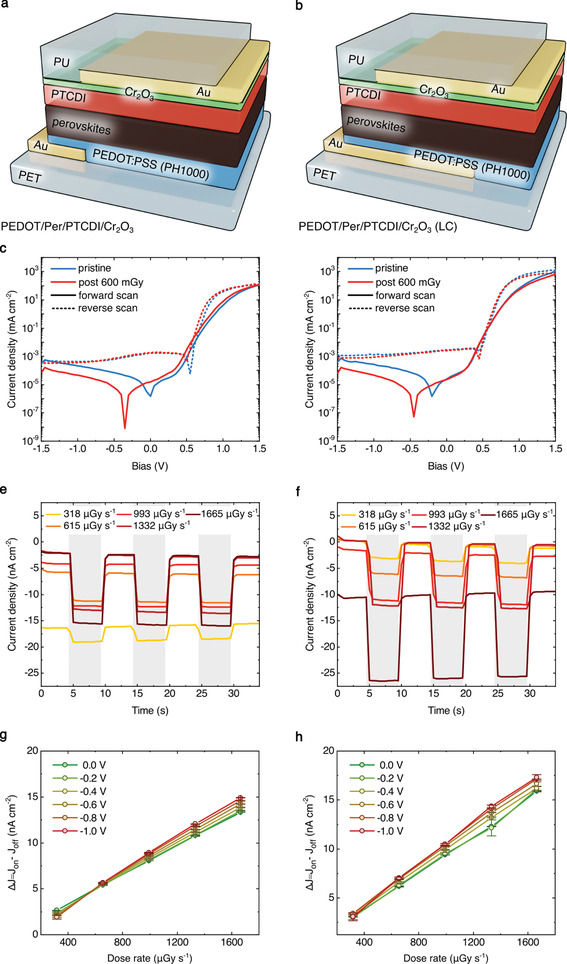
*N,N*′‐Dimethyl‐3,4,9,10‐perylentetracarboxylic diimide (PTCDI) based perovskite X‐ray detectors. Device architecture of X‐ray photodiodes containing PTCDI/Cr_2_O_3_/Au on the top side and a) poly(3,4‐ethylenedioxythiophene):poly(styrenesulphonate) (PEDOT:PSS), or b) PEDOT:PSS with Au electrode covering an entire pixel (LC, large contact). c,d)*J*−*V*characteristics of the two structures in dark conditions, before and after the X‐ray exposure. e,f) Dynamic detector response to three cycles of 5 s ON (gray boxes), 5 s OFF the X‐ray irradiation (40 kVp) at different incident dose rate (318, 615, 993, 1332, and 1665 μGy s^−1^). g,h) X‐ray induced photocurrent as a function of radiation incident dose rates, for reverse biases between 0 and −1 V. X‐ray photodiode with architecture PEDOT/Per/PTCDI/Cr_2_O_3_(LC) shows the best response amongst all the devices tested.

We fabricate a set of PTCDI‐based devices with a thin 10 nm Cr_2_O_3_ interlayer between ETL and Au electrode, using PEDOT:PSS (**Figure** **3**a,b) as HTL. In order to verify the active layer composition and phase purity we report XRD data, showing characteristic diffraction peaks of tetragonal perovskites (Figure S6a, Supporting Information). Device structure is corroborated by cross‐sectional SEM image, as well as energy‐dispersive X‐ray spectroscopy (EDX) maps and spectra confirming the elemental composition of the device layers (Figure S6b−d, Supporting Information). Similar to the PCBM‐based set, devices in Figure 3b use large metal bottom contacts to improve charge extraction and overall device performance. It has been demonstrated that a Cr_2_O_3_ interfacial layer improves the performance of perovskite and organic solar cells, due to its hole blocking capabilities (CB  =  4.0 eV, *E*_g_  =  3.4 eV).^[^ ^62^ ^]^ Moreover, its chemical resistance effectively shields commonly used metal contacts from detrimental reactions with oxidizing and halide‐forming iodide species, making the devices more stable in air.^[^ ^44^ ^]^ Thermal evaporation of PTCDI excellently covers the perovskite absorber layer and results in 100% device yield, nevertheless this type of detector architecture shows electrical hysteresis, as evident from *J*−*V* curves in Figure 3c,d. We observe, that all PTCDI‐based detectors show box‐like highly reproducible response, although current hysteresis in *J*−*V* measurements translates into a shift in the dark current that can be observed during the dynamic response measurements over three ON/OFF cycles with dose rates between 318 and 1665 μGy s^−1^ (Figure 3e,f). Moreover, X‐ray response stability for multiple cycles of irradiation is confirmed in a dynamic measurement lasting 21 consecutive cycles at dose rate of 52 mGy s^−1^, while sweeping the applied bias between 0 and −1.2 V (Figure S7, Supporting Information).

During X‐ray photocurrent density (Δ*J*) measurements we note a linearly increasing signal as a function of increasing dose rate in PTCDI‐based devices (Figure 3g,h), with a moderate voltage dependence, comparable to a PCBM‐based set. The PEDOT/Per/PTCDI/Cr_2_O_3_ architecture results in devices with sensitivity of 7.9 ± 0.4 µC Gy^−1^ cm^−2^, comparable to the best performing architecture of the PCBM‐based set. The use of large bottom metal contacts in the PEDOT/Per/PTCDI/Cr_2_O_3_ (LC) architecture further increases charge collection efficiency, resulting in 9.3 ± 0.5 µC Gy^−1^ cm^−2^ sensitivity, the current record for thin film perovskite X‐ray detector operating at 0 V.^[^ ^38^ ^]^ The estimation of further detector parameters such as UV−vis external quantum efficiency, X‐rays effective efficiency, responsivity, and photoconductive gain are reported in Table S3 and Figure S8 (Supporting Information).

### 2.4 Free‐Standing Isotropic Devices

**Figure 4:**
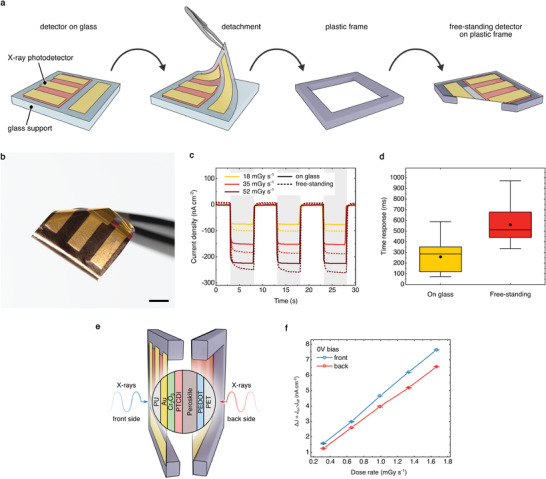
Free standing ultraflexible X‐ray detector. a) Schematic of handling and detaching X‐ray photodetectors fabricated on 1.4 µm PET substrates from their glass support and transferring them onto a plastic frame for mechanical support needed during the measurements. b) Photograph of the ultraflexible X‐ray detector held with tweezers after detaching (scale bar 5 mm). c) Comparison of the dynamic response between the detector on the glass support (continuous lines) and the same device in the free‐standing configuration (dotted lines) at different dose rates (18, 35, and 52 mGy s^−1^). Free‐standing devices show slower response time, but improved detection performance. d) Time response difference between on‐glass and free‐standing detectors. e) Schematic illustration of the front and back side configurations of the isotropic X‐ray detection measurement. f) Photocurrent versus dose rate plot for X‐rays impinging from the back (red) and from the front (blue) of the detector. Using ultrathin substrates for X‐ray photodetector fabrication allows for comparable device performance regardless of the side from which the X‐ray radiation is impinging.

The possibility to make extremely thin, ultraflexible perovskite‐based X‐ray photodetectors is a distinctive advantage of using a 1.4 µm PET foil substrates. Here, we investigate reliability and performance of free‐standing devices in PEDOT/Per/PTCDI/Cr_2_O_3_ (FS) architecture. In order to simplify the device handing, we transfer the free‐standing detectors from their glass support onto a plastic carrier frame as shown in **Figure** **4**a. Figure 4b shows an ultraflexible detector held with tweezers after detaching it from the glass support. We observe a sensitivity of 7.3 ± 0.3 µC Gy^−1^ cm^−2^ at 0 V, which is comparable to the value recorded for the same sample architecture on glass support, 7.9 ± 0.4 µC Gy^−1^ cm^−2^ (Figure 1c). The exceptionally low limit of detection, down to 1.7 ± 0.2 μGy s^−1^, allows to envisage the employment of such detectors for space, medical dosimetry, and diagnostic application, where the exposure of the patient must be kept as low as possible. Figure 4c shows the comparison of dynamic response between the detector on glass support and in free‐standing form to three cycles of X‐ray irradiation at three different dose rates (18, 35, and 52 mGy s^−1^). Interestingly, the free‐standing X‐ray photodiode photocurrent amplitude does not degrade, but rather slightly increases (Figure S9, Supporting Information). Furthermore, the sensitivity of free‐standing detectors exhibits a more pronounced dependence to the applied bias, surpassing its glass supported counterpart at biases higher than 0.3 V (Figure S2, Supporting Information). The enhanced bias‐dependent X‐rays response can be ascribed to the activation of a photoconductive gain process assisted by the defective states induced in the detector by the mechanical strain (in perovskite and/or in the organic semiconductor). This interpretation is supported by the slower response time for free‐standing devices (Figure 4d). In fact, gain is usually observed when the carrier lifetime exceeds the carrier transit time, and thus high gain is often associated with long carrier trapping,^[^ ^63^, ^64^ ^]^ which results in slower detector response.^[^ ^10^, ^37^ ^]^

Additionally, we evaluate the performance of the ultraflexible PTCDI‐based device under repeated bending cycles with bending radius as small as 0.25 mm (Figure S10a, Supporting Information). The *J−V* curves measured before and after up to 80 bending cycles show very small changes in the reverse bias region (Figure S10b, Supporting Information). Reproducible and steady performance is also observed during dynamic measurements of dark current while bending the device (Figure S10c, Supporting Information). Robustness of these ultraflexible devices can be further understood by analyzing their ultrathin architecture and design which places the neutral mechanical plane (zero strain plane) within the active region of the X‐ray photodiode and thus reducing the mechanical stress that the perovskite layer experiences (Figure S10d, Supporting Information).

We observe that using 1.4 µm thin and low absorbing PET substrates (see material attenuation fractions Figure S3, Supporting Information) for ultraflexible perovskite X‐ray detectors fabrication allows for the unique feature of isotropic operation (Figure 4e). In fact, due to their similar chemical composition and density, the X‐rays attenuated fraction between PET substrate and the PU encapsulation layer is comparable (they differ of about 0.01%, at 15.2 keV, i.e., the mean photon energy of the radiation emitted by the W‐target X‐ray tube at 40 kVp). This similarity is shown in Figure S11 (Supporting Information), reporting the plot of the attenuated fraction for 1.4 µm PET layer and 1 µm thick PU layer in function of the photon energy. In other words, the intensity of the X‐rays impinging onto the device from the two sides is only slightly different.

Thus, our free‐standing ultrathin perovskite X‐ray detectors show comparable photocurrent response to ionizing radiation impinging either on the front (mechanical protection PU layer side) or back (PET substrate side) of the device (Figure 4f). The sensitivity of these detectors also increases linearly with the dose rate with slightly higher values for front side irradiation and comparable bias dependence for both sides (Figure S12, Supporting Information). Isotropic X‐ray detectors are of special interest to applications where the direction of the incoming radiation is not known, thus allowing for reliable, real‐time detection of ionizing radiation without the need to align or orient the device.

## 3 Conclusion

Here, we demonstrate the thinnest and most flexible perovskite X‐ray detectors achieved to date. Several perovskites photodiode/solar cell ETL and HTL materials were used to examine device performance through interface engineering. We achieve fully passive thin film perovskite X‐ray detectors with a sensitivity of 9.3 ± 0.5 µC Gy^−1^ cm^−2^ operated at 0 V (PEDOT/Per/PTCDI/Cr_2_O_3_ (LC)), dark current as low as 0.030 ± 0.004 nA cm^−2^ (NiO*_x_*/Per/PCBM/BCP) and limit of detection of 0.58 ± 0.05 μGy s^−1^ (PEDOT/Per/PCBM/TiO*_x_*). The ultraflexible X‐ray detectors show comparable performance in the free‐standing form to their on‐glass substrate counterparts. Furthermore, these devices can detect X‐ray irradiation equally well from both front and back side. Isotropic detection of X‐ray radiation is a unique feature arising due to the ultrathin nature of these devices and is unattainable for other detector architectures relying on thick substrates.

The here reported ultraflexible X‐ray detectors present a number of definite advancements over previously existing technology. The extremely low weight and practically imperceptible nature of these devices opens a platform for further development of ionizing radiation detectors for use in extreme environments such as outer space or disaster relief sites, where carried load is a premium. The intrinsic conformability of ultraflexible X‐ray detectors to any elaborate surface will benefit not only medical imaging, but also nondestructive testing in an industrial setting. Moreover, our devices have a high potential for industrial scale‐up through integration into large matrices where both interconnects and detectors can withstand severe mechanical stresses. Solar cells and other electronics built on ultrathin plastic foils confirm that these ultraflexible X‐ray detectors can operate with high resilience and reliability. Devices with comparable thickness fabricated on 1.4 µm PET foil have shown to accommodate bending radii down 10 µm, enduring crumpling, wrinkling, and other wear.^[^ ^43^, ^44^, ^65^ ^]^

Future work should focus on fundamental aspects, investigating the influence of different perovskite compositions on X‐ray photodiode performance, as well as integration into matrices for imaging, and thorough examination of prolonged mechanical stresses onto the device performance.

## 4 Experimental Section

##### Materials

All chemicals and solvents were purchased from commercial suppliers and used as received, if not stated otherwise. Hellmanex III detergent (Hellma Analytics), Sylgard 184 Silicone Elastomer (PDMS, Dow Corning), PET foil (Mylar CW02), hexane (*n*‐hexane, VWR, 98%), PEDOT:PSS aqueous dispersion (Clevios PH1000, Heraeus), Zonyl FS‐300 (abcr GmbH), nickel chloride hexahydrate (NiCl_2_⋅6H_2_O, Sigma Aldrich, 99.9%), sodium hydroxide (NaOH, Sigma Aldrich, ≥98%), lead iodide (PbI_2_, Sigma Aldrich, 99.9%), lead bromide (PbBr_2_, Sigma Aldrich, 99.99%), cesium iodide (Sigma Aldrich, 99.9%), *N*,*N*‐dimethylformamide (DMF, anhydrous, Sigma Aldrich), dimethylsulfoxide (DMSO, VWR, 99.5%), chlorobenzene (VWR, reagent grade), [6,6]−phenyl‐C61‐butyric acid methyl ester (PCBM, Solenne BV), chloroform (VWR, 99.2%), titanium (IV) isopropoxide (Ti[OCH(CH_3_)_2_]_4_, Sigma Aldrich, 99.9+%), 2‐methoxyethanol (CH_3_OCH_2_CH_2_OH, Sigma Aldrich, 99.9%), ethanolamine (H_2_NCH_2_CH_2_OH, Sigma Aldrich, 99%), isopropanol (2‐propanol, reagent grade), bathocuproine (BCP, Sigma Aldrich, 96 %), *N*,*N*′‐dimethyl‐3,4,9,10‐perylentetracarboxylic diimide (PTCDI, purified through sublimation 2 times, Hoechst), UV curable epoxy (E131 encapsulation epoxy, Ossila), PP foil, and PU resin(CRC Kontakt Chemie Urethan 71).

Methylammonium bromide (MABr) was synthesized from methylamine [33 weight% (wt%) in absolute ethanol; Sigma Aldrich] and hydrobromic acid (HBr, 48 wt%, aqueous; Sigma Aldrich) and purified using diethylether (VWR) and absolute ethanol (Merck Millipore) as described in literature.^[^ ^66^, ^67^ ^]^ Methylammonium iodide (MAI) and formamidinium iodide (FAI) were synthesized using analogous procedure using hydroiodic acid (HI, 57 wt%, aqueous; Sigma Aldrich).

##### Solution Preparation

PDMS solution was prepared by mixing 1:10 w/w of cross‐linker to hardener and then diluting it 1:1 w/w with hexane.

PEDOT:PSS solution was prepared by mixing Clevios PH1000 stock solution with 5 vol% DMSO and 0.5 vol% Zonyl FS‐300, stirring at room temperature for an hour and keeping at 4 °C overnight. PEDOT:PSS solution was filtered through Minisart RC25 Syringe filter 0.45 µm regenerated cellulose right before use. NiO*_x_* nanoparticles were prepared based on a procedure reported by Hailegnaw et al.^[^ ^55^ ^]^

Perovskite solution (Cs_0.05_(FA_0.83_MA_0.17_)_0.95_PbI_3‐_ *_x_*Br*_x_*) was prepared by mixing PbI_2_ (507.5 mg, 1.10 mmol), FAI (172 mg, 1.00 mmol), MABr (22.4 mg, 0.20 mmol), and PbBr_2_ (73.5 mg, 0.20 mmol) in 1 mL of DMF and DMSO (4:1 v/v ratio, respectively) followed by stirring at 45 °C until dissolved. Afterwards, CsI (≈0.063 mmol, from 1.5 m stock solution in DMSO) was added to the mixture and stirred overnight.^[^ ^55^ ^]^ The solution was filtered using polytetrafluoroethylene (PTFE) syringe filters (0.45 µm; Whatman) before spin‐coating.

PCBM solution was prepared by dissolving 2 wt% PCBM in chlorobenzene and chloroform (1:1 volume ratio). BCP solution was prepared in concentration of 0.5 mg mL^−1^ in isopropanol. TiO*_x_* solgel was prepared based on procedure reported by Heilgenaw et al.^[^ ^55^ ^]^

##### Device Fabrication

Glass substrates (1.5 × 1.5 cm, 1 mm thick) were cut and cleaned in an ultrasonic bath for 30 min each in 2 v/v% Hellmanex in DI water solution, 2 × DI water solution, isopropanol, and dried using N_2_. Then PDMS solution was spin‐coated at 4000 rpm for 30 s on glass and placed on a heat plate at 150 °C for 10 min to cross‐link. Next, the 1.4 µm PET foil was carefully placed on the sample avoiding air pockets and then transferred to a heating plate again at 110 °C for another 10 min. Afterwards, Cr/Au (10/100 nm) bottom contacts were thermally evaporated (0.1–1 nm s^−1^ at base pressure ≈1  ×  10^−6^ mbar). The hole transport material (HTL) was PEDOT:PSS for device architecture PEDOT/Per/PCBM/BCP, PEDOT/Per/PCBM/TiOx, PEDOT/Per/PTCDI/Cr_2_O_3_, PEDOT/Per/PTCDI/Cr_2_O_3_ (LC), and NiO*_x_* for NiO*_x_*/Per/PCBM/BCP (LC). PEDOT:PSS solution was spin‐coated at 1500 rpm for 45 s (ramp 2 s) followed by 1000 rpm for 2 s (ramp 1 s) and annealed at 122 °C for 15 min. Then the film was washed by spin‐coating isopropanol solution at 1500 rpm for 4 s followed by 4000 rpm for 12 s and annealed at 120 °C for 15 min. NiO*_x_* film was obtained by spin‐coating the dispersion at 4000 rpm for 15 s and then 5000 rpm for 15 s. Afterwards the film was annealed at 140 °C for 20 min. For further deposition of the perovskite layer the samples were transferred into N_2_ glovebox. Perovskite solution was deposited using anti‐solvent procedure. The solution was spin‐coated in two steps at 1500 rpm for 10 s with ramp 150 rpm s^−1^ followed by 6000 rpm for 30 s with ramp 3000 rpm s^−1^. Approximately ≈0.2 mL of chlorobenzene (anti‐solvent) was dropped at 23rd second for about 3 s. Then the film was annealed at 100 °C for 1 h. The electron transporting materials (ETL) were PCBM for samples of the first set (PEDOT/Per/PCBM/BCP, PEDOT/Per/PCBM/TiO*_x_*, NiO*_x_*/Per/PCBM/BCP (LC)) and PTCDI for samples of the second set (PEDOT/Per/PTCDI/Cr_2_O_3,_ PEDOT/Per/PTCDI/Cr_2_O_3_ (LC)).

PCBM solution was spin‐coated onto sample at 1300 rpm for 16 s (ramp 2 s) followed by 2000 rpm for 15 s (ramp 2 s). BCP was spin‐coated at 5000 rpm for 30 s on sample PEDOT/Per/PCBM/BCP and NiO*_x_*/Per/PCBM/BCP (LC) and TiO*_x_* was spin‐coated at 4000 rpm 30 s (ramp 2 s) on sample PEDOT/Per/PCBM/TiO*_x_* and annealed at 110 °C  for about 5 min in ambient atmosphere. Al contacts were then thermally evaporated on all PCBM containing samples at rate of 0.01−0.5 nm s^−1^ and base pressure ≈3  ×  10^−6^ mbar. These devices then were encapsulated using PP foil and UV curable epoxy.

PTCDI layer (100 nm) was deposited onto sample via thermal evaporation at 0.5–2 nm s^−1^ rate and base pressure ≈1  ×  10^−6^ mbar. This was followed by thermal evaporation of Cr/Au contacts (10/100 nm) at rate of 0.1–1 nm s^−1^ and base pressure ≈1  ×  10^−6^ mbar. Finally, these devices were encapsulated by spin‐coating PU at 1000 rpm for 30 s. PU layer was cross‐linked at room temperature for about 24 h.

Ultraflexible X‐ray photodetectors were lifted from their glass support using transfer printing technique with Parafilm as the carrier in order to provide additional mechanical support and reduce bending stress during transfer. Resulting photodiodes were fixed to a plastic PET frame using double‐sided Kapton tape allowing to handle them in their freestanding form.

##### Device Characterization under X‐Rays

Electrical characterization of the samples under X‐ray irradiation was performed in a N_2_‐filled faraday box with a 70 µm front Al window. Two different X‐ray sources were used to address different incident dose rate conditions. Hamamtsu L12161 X‐ray tube with tungsten target was used at fixed 40 kV operating voltage the filament current was changed between 100 and 500 µA leading to an incident dose rate on the samples between 318 and 1665 μGy s^−1^. The other X‐ray tube used was a Mo target PANalytical PW2285/20, with a beryllium window, used at 35 kV with a current between 10 and 30 mA and respective dose rates between 18 and 52 μGy s^−1^. The dose rate calibrations were previously performed employing the Barracuda radiation detector (RTI Group). The modulation of the beam was obtained with a mechanical lead shutter placed close to the X‐ray tube window. Keithley SMU 2614 was used in combination with a LabVIEW program for electrical signal acquisition.

##### Flexibility Characterization

Bending tests were performed on PEDOT/Per/PTCDI/Cr_2_O_3_ device using in‐house built uniaxial stretcher in combination with a Python program for position control (actuator speed 1 mm s^−1^) and electrical signal acquisition from Keithley 2600.

##### Material Characterization

Zeiss 1540 XB CrossBeam scanning electron microscope equipped with OXFORD Instruments EDX system was used to perform scanning electron microscopy (SEM) (acceleration voltage 5 keV) EDX analysis (acceleration voltage 10 keV).

X‐ray diffraction (XRD) analysis was performed using Bruker D8 XRD system employing Cu and K*α* radiation source (*λ* = 1.5418 nm at 40 kV and 20 mA). A PerkinElmer Lambda 1050, UV/Vis/NIR spectrometer was used to measure the transmission spectrum of the perovskite film.

## Conflict of Interest

The authors declare no conflict of interest.

## Supporting information

Supporting Information
