# Supplementary material for: Designing Ultraflexible Perovskite X‐Ray Detectors through Interface Engineering
Source: Adv Sci (Weinh). 2020 Nov 13;7(24):2002586. doi: 10.1002/advs.202002586 (PMC7740104; doi:10.1002/advs.202002586)
Supplement: Supplementary file 1 — Supporting Information [file ADVS-7-2002586-s001.pdf]

WILEY-VCH

© 2020 Wiley-VCH GmbH

ADVANCED SCIENCE
Open Access

Supporting Information

for *Adv. Sci.,* DOI: 10.1002/advs.202002586

Designing Ultraflexible Perovskite X-ray Detectors through Interface Engineering

Stepan Demchyshyn, Matteo Verdi, Laura Basiricò, Andrea Ciavatti, Bekele Hailegnaw, Daniela Cavalcoli, Markus Clark Scharber, Niyazi Serdar Sariciftci, Martin Kaltenbrunner*, Beatrice Fraboni*

Supporting Information

Designing ultraflexible perovskite X-ray detectors through interface engineering

Stepan Demchyshyn, Matteo Verdi, Laura Basiricò, Andrea Ciavatti, Bekele Hailegnaw, Daniela Cavalcoli, Markus Clark Scharber, Niyazi Serdar Sariciftci, Martin Kaltenbrunner*, Beatrice Fraboni*

Table S1: Comparison between perovskite film-based direct X-ray detectors reported in literature in the last years.

Material	Thickness	Flexibility assessment	V_{Bias}	S_A [μC Gy_{air}^{-1} cm^{-2}]	LoD [μGy_{air} s^{-1}]	X-ray Source	REF
$MAPbI_3$	260-600 nm	X	0V	0.4 - 1.5[a)]	NA	W-target, 75 kVp	S. Yakunin, M. Sytnyk, D. Kriegner, S. Shrestha, M. Richter, G. J. Matt, H. Azimi, C. J. Brabec, J. Stangl, M. V. Kovalenko, W. Heiss, Nat Photon 2015, 9, 444.
	60 μm		80 V	NA	NA	Cu-target (Kα 8keV)	
CsFAMA	450 nm	X	0 V<br>0.4 V	3.7<br>98	NA	Mo-target, 35 kVp (peak@17keV)	L. Basiricò, S. P. Senanayak, A. Ciavatti, M. Abdi- Jalebi, B. Fraboni, H. Sirringhaus, Advanced Functional Materials 2019, 29, 1902346.
	NA		10V	3.2	NA		
$MAPbI_3$	700 μm - 1 mm	X	0.2 V μm^{-1}	2527	48	W-target, 70 kVp (peak@38keV)	S. Shrestha, R. Fischer, G. J. Matt, P. Feldner, T. Michel, A. Osvet, I. Levchuk, B. Merle, S. Golkar, H. Chen, S. F. Tedde, O. Schmidt, R. Hock, M. Rührig, M. Göken, W. Heiss, G. Anton, C. J. Brabec, Nature Photonics 2017, 11, 436.
$MAPbI_3$	830 μm	X	10 - 200 V	$0.3 - 3.8 \times 10^3$ (single pixel)<br>1.1×10^4	NA	W-target, 100 kVp 3mmAl filter	Y. C. Kim, K. H. Kim, D.-Y. Son, D.-N. Jeong, J.-Y. Seo, Y. S. Choi, I. T. Han, S. Y. Lee, N.-G. Park, Nature 2017, 550, 87.
$CsPbBr_3$	240 μm	X	5 V mm^{-1}	5.57×10^4	0.215	W-target, 50 kVp (peak @30keV)	W. Pan, B. Yang, G. Niu, K.-H. Xue, X. Du, L. Yin, M. Zhang, H. Wu, X.-S. Miao, J. Tang, Advanced Materials 2019, 31, 1904405.
$CsPbBr_3$ QDs	20 nm	✓	0.1 V	83<br>17.7 (flex)	17.2	synchrotron radiation (100–2500 eV) Cu-target (peak @8 keV)	J. Liu, B. Shabbir, C. Wang, T. Wan, Q. Ou, P. Yu, A. Tadich, X. Jiao, D. Chu, D. Qi, D. Li, R. Kan, Y. Huang, Y. Dong, J. Jasieniak, Y. Zhang, Q. Bao, Advanced Materials 2019, 31, 1901644.
$CsPbBr_3$	18 μm	X	0 V<br>0.11V	470<br>1.7×10^3	0.053	Cu-target, 35kVp	Z. Gou, S. Huanglong, W. Ke, H. Sun, H. Tian, X. Gao, X. Zhu, D. Yang, P. Wangyang, physica status solidi (RRL) – Rapid Research Letters 2019, 13, 1900094.

$CsPbBr_3$	NA	X	5 V	NA	6×10^3 µGy	LINAC 6 MV and 25 MV	M. Bruzzi, C. Talamonti, N. Calisi, S. Caporali, A. Vinattieri, APL Materials 2019, 7, 051101.
Cs_2TeI_6	25 µm	X	1 V (250 $V\cdot cm^{-1}$)	19.2	NA	40 kVp	Y. Xu, B. Jiao, T.-B. Song, C. C. Stoumpos, Y. He, I. Hadar, W. Lin, W. Jie, M. G. Kanatzidis, ACS Photonics 2019, 6, 196.
$CsPbBr_3$	250 µm	X	300 V	1450	0.5	70 kVp 2mm Al filtered	G. J. Matt, I. Levchuk, J. Knüttel, J. Dallmann, A. Osvet, M. Sytnyk, X. Tang, J. Elia, R. Hock, W. Heiss, C. J. Brabec, Advanced Materials Interfaces n.d., n/a, 1901575.
CsFAMA	3.7 µm	✓	0.1 V	59.9	12	W-target 70 kVp 0.4 mm Al filter	H. Mescher, F. Schackmar, H. Eggers, T. Abzieher, M. Zuber, E. Hamann, T. Baumbach, B. S. Richards, G. Hernandez-Sosa, U. W. Paetzold, U. Lemmer, ACS Appl. Mater. Interfaces 2020, 12, 15774.
$MAPbI_3$	800 µm	X	1 V<br>10 V	2.20×10^4<br>1.22×10^5	NA	Ag-target, 40kVp	M. Hu, S. Jia, Y. Liu, J. Cui, Y. Zhang, H. Su, S. Cao, L. Mo, D. Chu, G. Zhao, K. Zhao, Z. Yang, S. F. Liu, ACS Appl. Mater. Interfaces 2020, 12, 16592.
$(BA)_2(MA)_2Pb_3I_{10}$ (Pb_3)	470 nm	X	0 V	13	400	Synchrotron radiation 8.05 and 10.9 keV	H. Tsai, F. Liu, S. Shrestha, K. Fernando, S. Tretiak, B. Scott, D. T. Vo, J. Strzalka, W. Nie, Science Advances 2020, 6, eaay0815.
CsFAMA	500 nm	✓	0 V	9.3	0.5	W-target, 40 kVp	THIS WORK

Table S2: Performance characteristic summary of the device architectures prepared during this study. LC – Large Contact, FS – Free Standing film.

**Device structure**	**Normilized sensitivity** ($\mu C\ Gy^{-1}\ cm^{-2}$)	**Dark current** ($nA\ cm^{-2}$)	**Limit of deteciton** ($\mu Gy\ s^{-1}$)
PEDOT/Per/PCBM/BCP	2.15±0.08	2.7±0.1	2.5±0.4
PEDOT/Per/PCBM/TiOx	7.5±0.3	1.62±0.06	0.58±0.05
NiOx/Per/PCBM/BCP (LC)	3.0±0.2	0.030±0.004	2.5±0.3
PEDOT/Per/PTCDI/Cr_2O_3	7.9±0.4	2.1±0.1	9.9±0.2
PEDOT/Per/PTCDI/Cr_2O_3 (LC)	9.3±0.5	0.5±0.2	24.1±0.6
PEDOT/Per/PTCDI/Cr_2O_3 (FS)	7.3±0.3	33±1	1.7±1.2

Table S3: Effective efficiency and responsivity to the X-rays of the here reported ultraflexible perovskite X-ray detectors. The effective efficiency, i.e. the charge collected over the total generated charges has been evaluated as defined in Ref.[6]. All the devices exceed 100%, demonstrating the efficient collection of charges by means of the internal electric field. To justify effective efficiency values greater than 100%, in addition to the direct collection of primary generated charges, a photoconductive gain mechanism process takes place with an amplification factor up to 8. For the calculation of the total charge generated under the exposure to X-rays, the simulated spectral energy distribution of the X-ray W-target at 40 kV, normalized by the measured total photon flux has been used. We considered e^{-}-h^{+} pair creation energy following the Klein's rule[58], $W_{\pm} \approx 2.8 \times E_{GCsFAMA} = 2.8 \times 1.54$ eV = 4.31eV

**Structure**	**Effective Efficiency (%)**	**Responsivity (mA/W)**
PEDOT/Per/PCBM/BCP	217%	5.0
PEDOT/Per/PCBM/TiOx	661%	13.5
NiOx/Per/PCBM/BCP (LC)	361%	8.9
PEDOT/Per/PTCDI/Cr_2O_3	711%	14.3
PEDOT/Per/PTCDI/Cr_2O_3 (LC)	820%	16.6

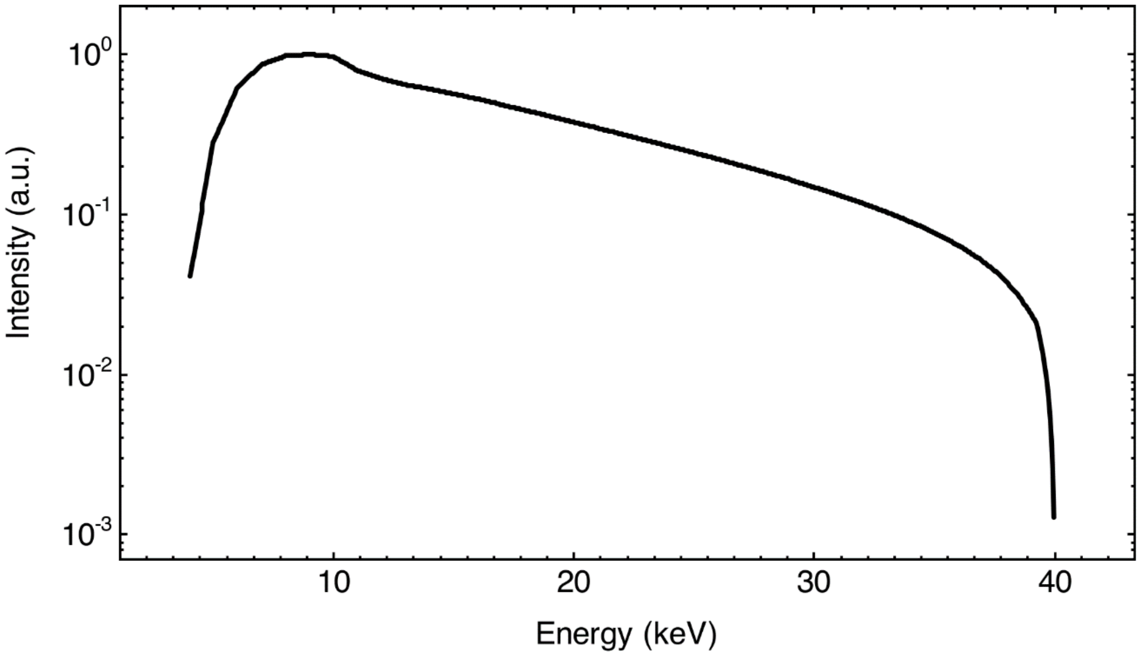

Figure S1: Energy spectrum of the W-target X-ray tube at 40 kVp. The mean energy of the spectrum is 15.2 keV.

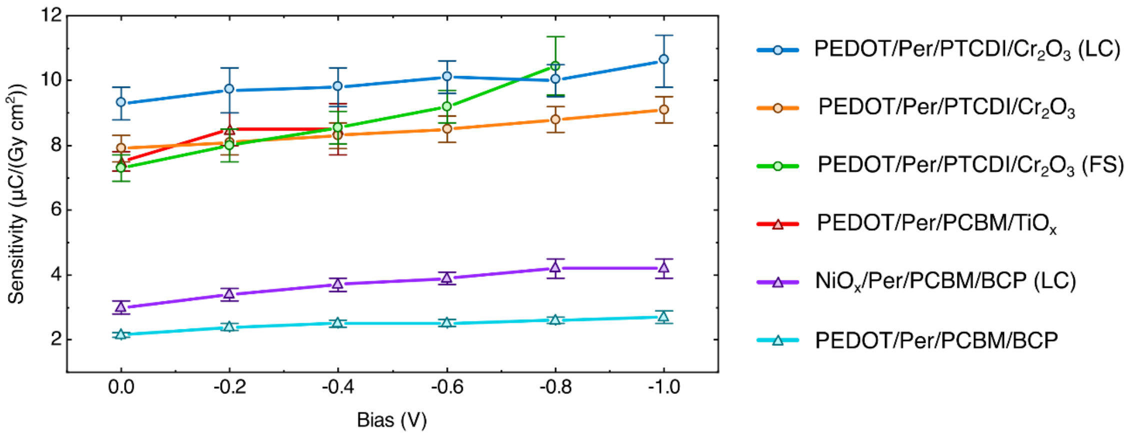

Figure S2: Plot of the sensitivity values at different biases for all the perovskite-based X-ray photodiodes. LC – large contact, FS – free-standing.

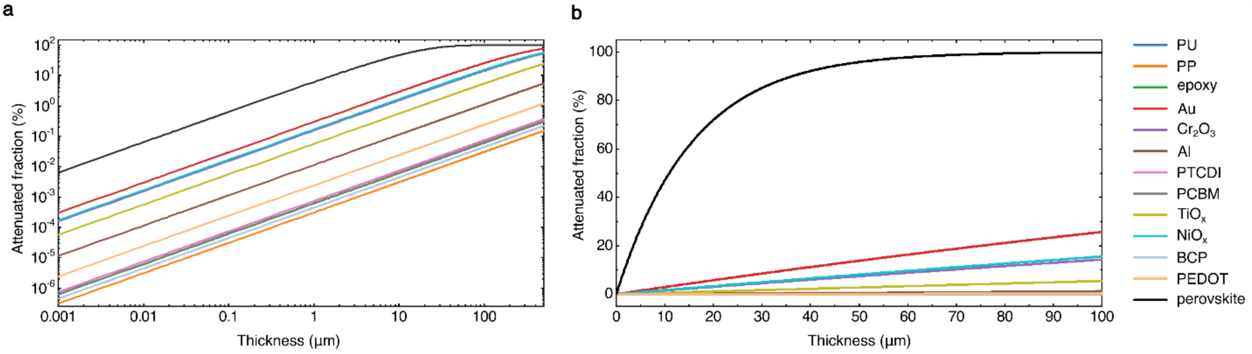

Figure S3: Attenuated fraction of materials used for fabrication of ultraflexible perovskite X-ray detectors **a,** on 1 µm and **b,** 100 µm scale.

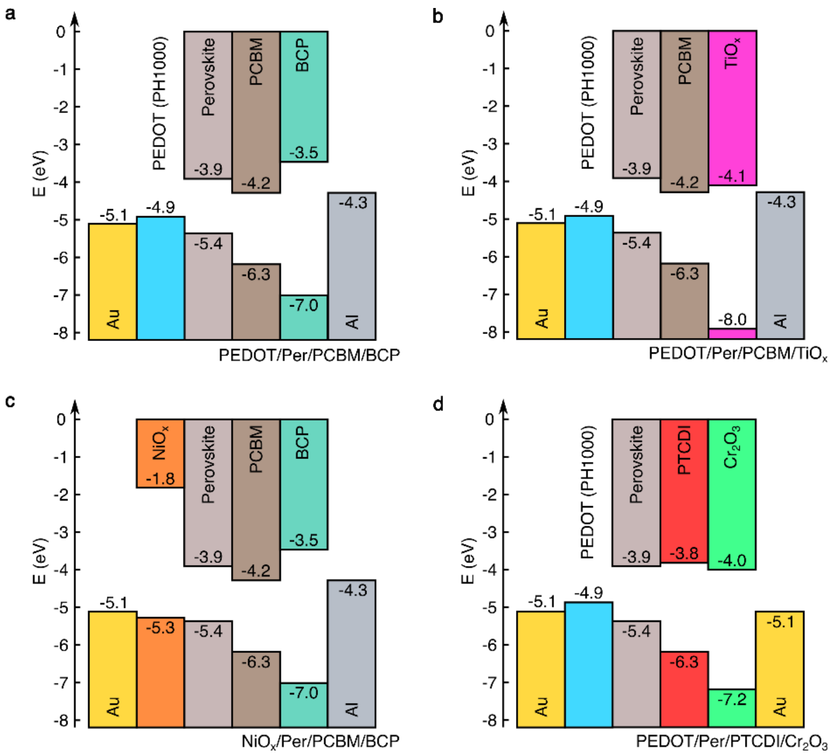

Figure S4: Energy band diagrams of perovskite X-ray photodiode architectures. PCBM-based **a,** PEDOT/Per/PCBM/BCP **b,** PEDOT/Per/PCBM/TiO_x **c,** NiO_x/Per/PCBM/BCP and PTCDI-based **d,** PEDOT/Per/PTCDI/Cr_2O_3 (applicable for both large contact (LC) and free-standing (FS) devices).

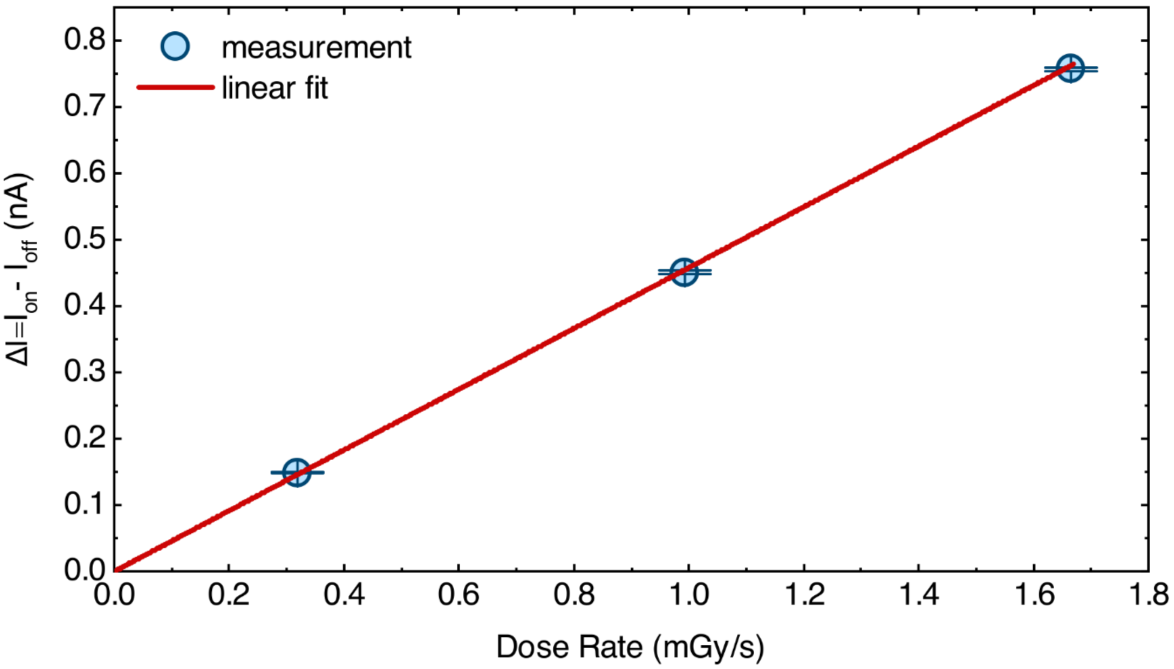

Figure S5: Plot of the photocurrent vs. dose date used for the estimation of PEDOT/Per/PCBM/TiO_x X-ray detectors limit of detection.

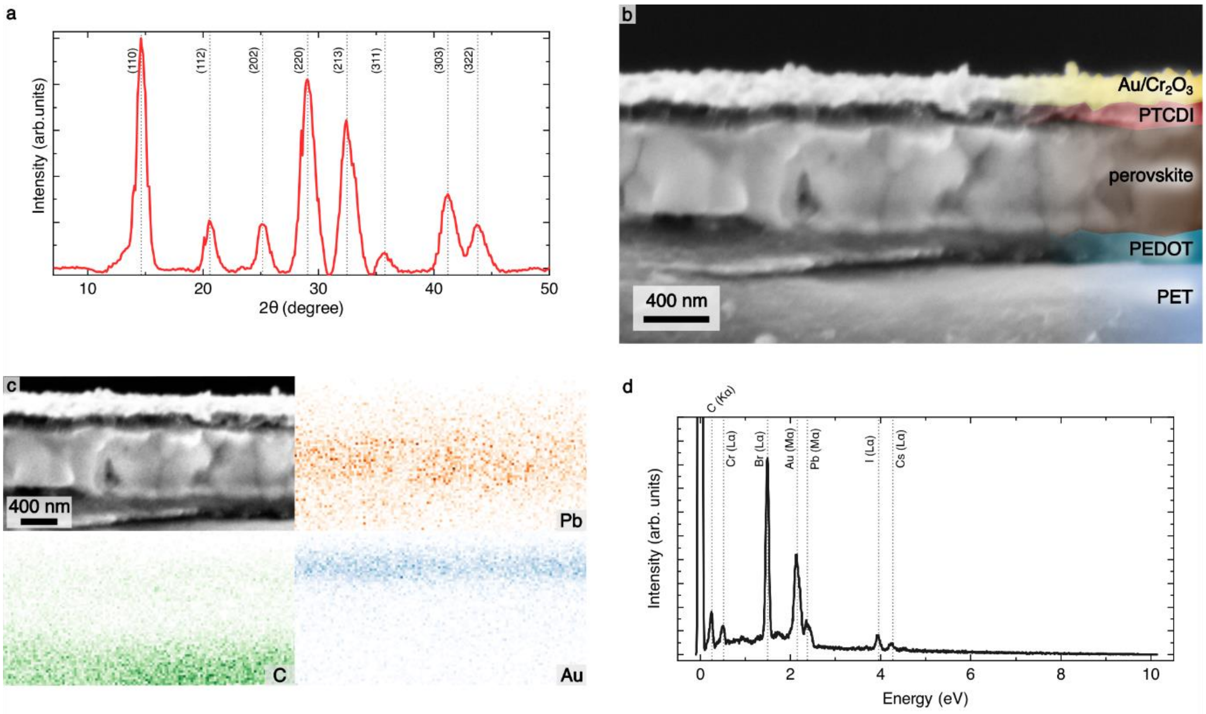

Figure S6: Structural and material characterization of PTCDI/Cr_2O_3/Au type X-ray photodiode. **a,** X-ray diffractogram obtained from mixed-cation mixed-halide perovskite thin film with characteristic tetragonal perovskite peaks indexed, verifying the composition of the active absorbing layer. **b,** Cross-section SEM image of the device showing ~ 500 nm perovskite active layer surrounded by charge transporting layers (PEDOT and PTCDI) and contact (Au/Cr_2O_3), highlighting its device architecture. **c,** EDX elemental map delineating and confirming device structure, with Pb, C, and Au traces identifying perovskite active layer, PEDOT and PET foil, as well as Au top contacts respectively. **d,** EDX spectrum showing peaks characteristic of carbon (PEDOT, PTCDI, PET), chromium and gold (Au/Cr_2O_3 top contacts), bromine, lead, iodine, and cesium (mixed-cation mixed-halide perovskite).

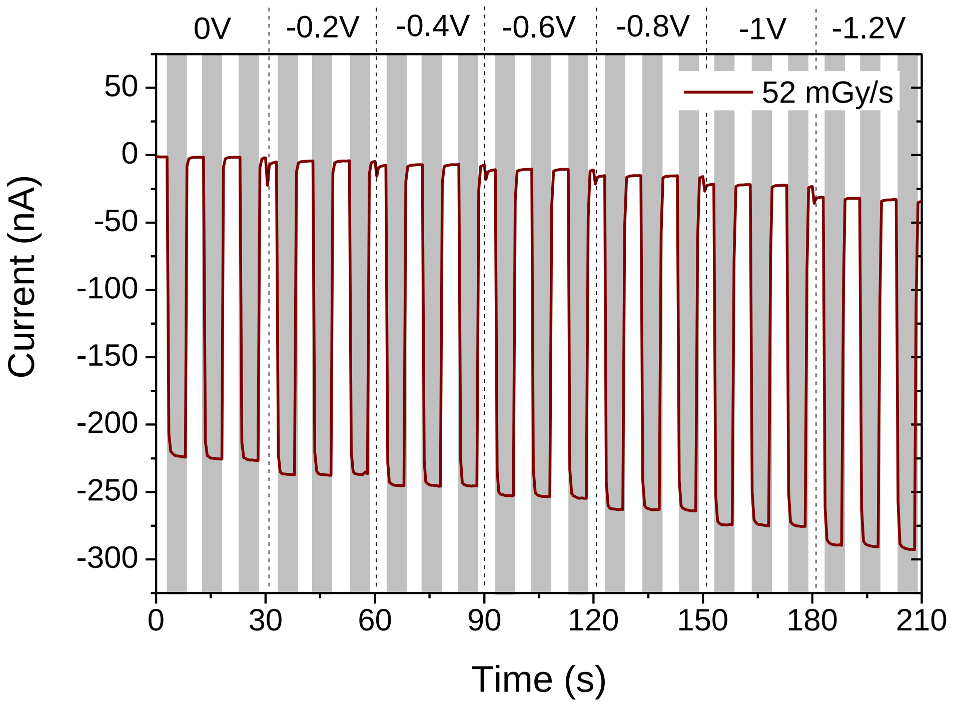

Figure S7: PTCDI-based detector's dynamic response to 21 consecutive X-rays irradiation cycles with a dose rate of 52 mGy/s, even while sweeping the applied bias between 0 and -1.2 V.

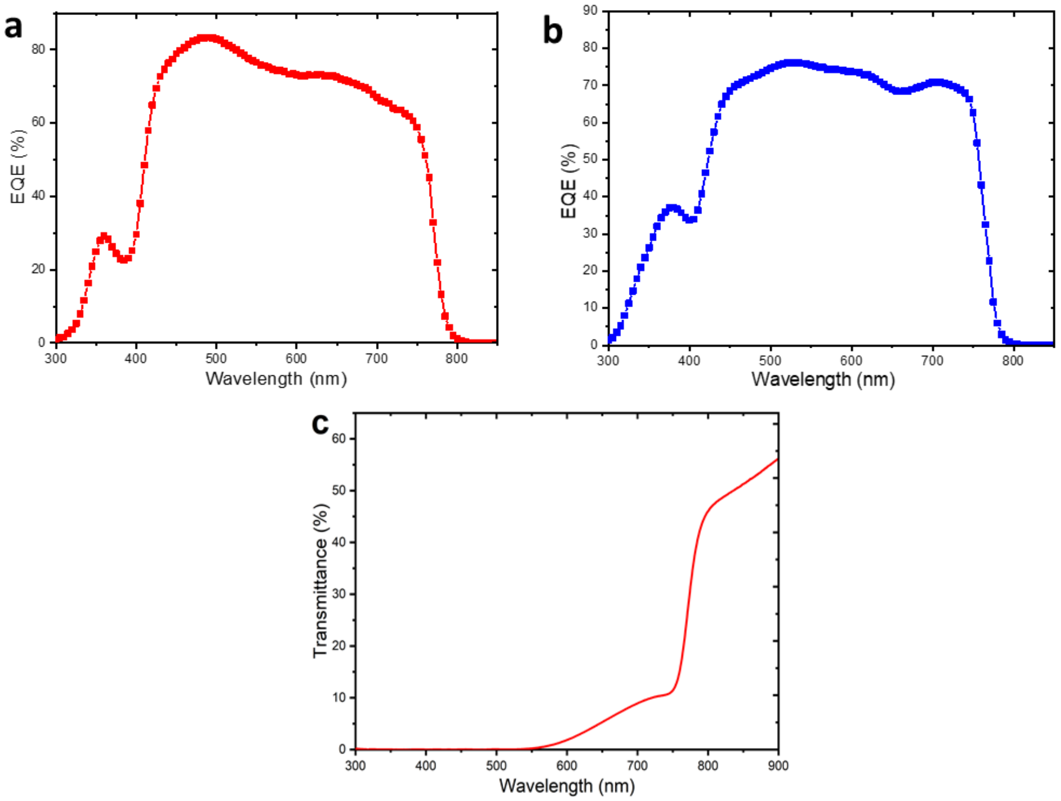

Figure S8: UV-vis external quantum efficiency (EQE) spectra of perovskite devices with (a) PTCDI and (b) PCBM/BCP electron transport layer and UV-Vis transmittance spectrum of mixed-cation mixed-halide perovskite film. The film shows strong absorbance in the UV-Vis region with onset absorbance around 800 nm. The transmittance loss above 800 nm is related to scattering and reflection loss of radiation in the film and across the glass substrate.

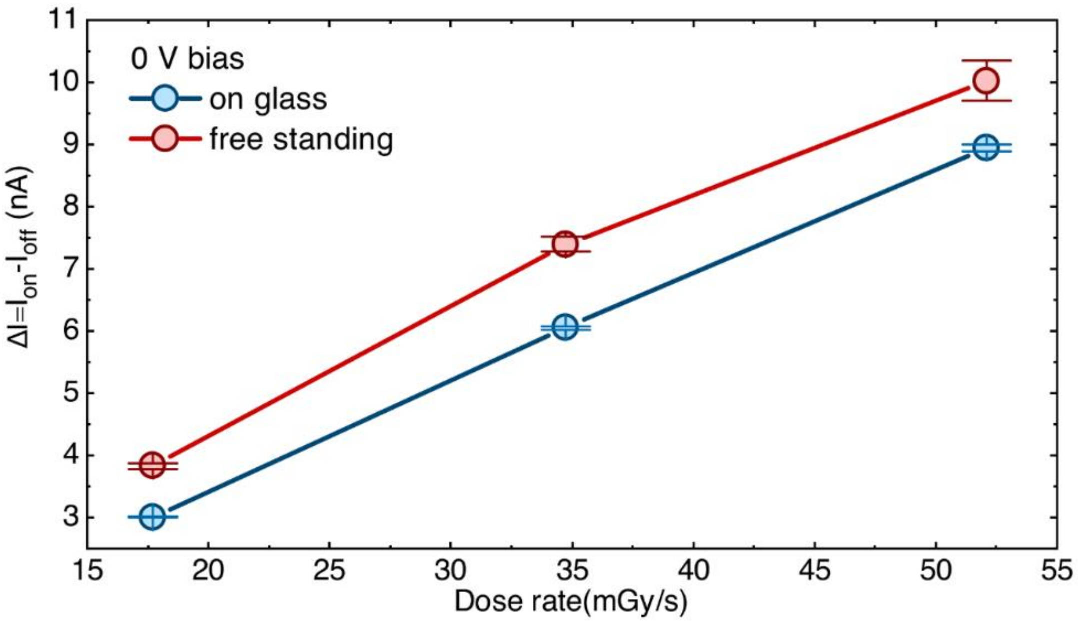

Figure S9: X-ray induced photocurrent as function of dose rate for the PEDOT/Per/PTCDI/Cr_2O_3 detector on glass carrier (blue) and free-standing (red).

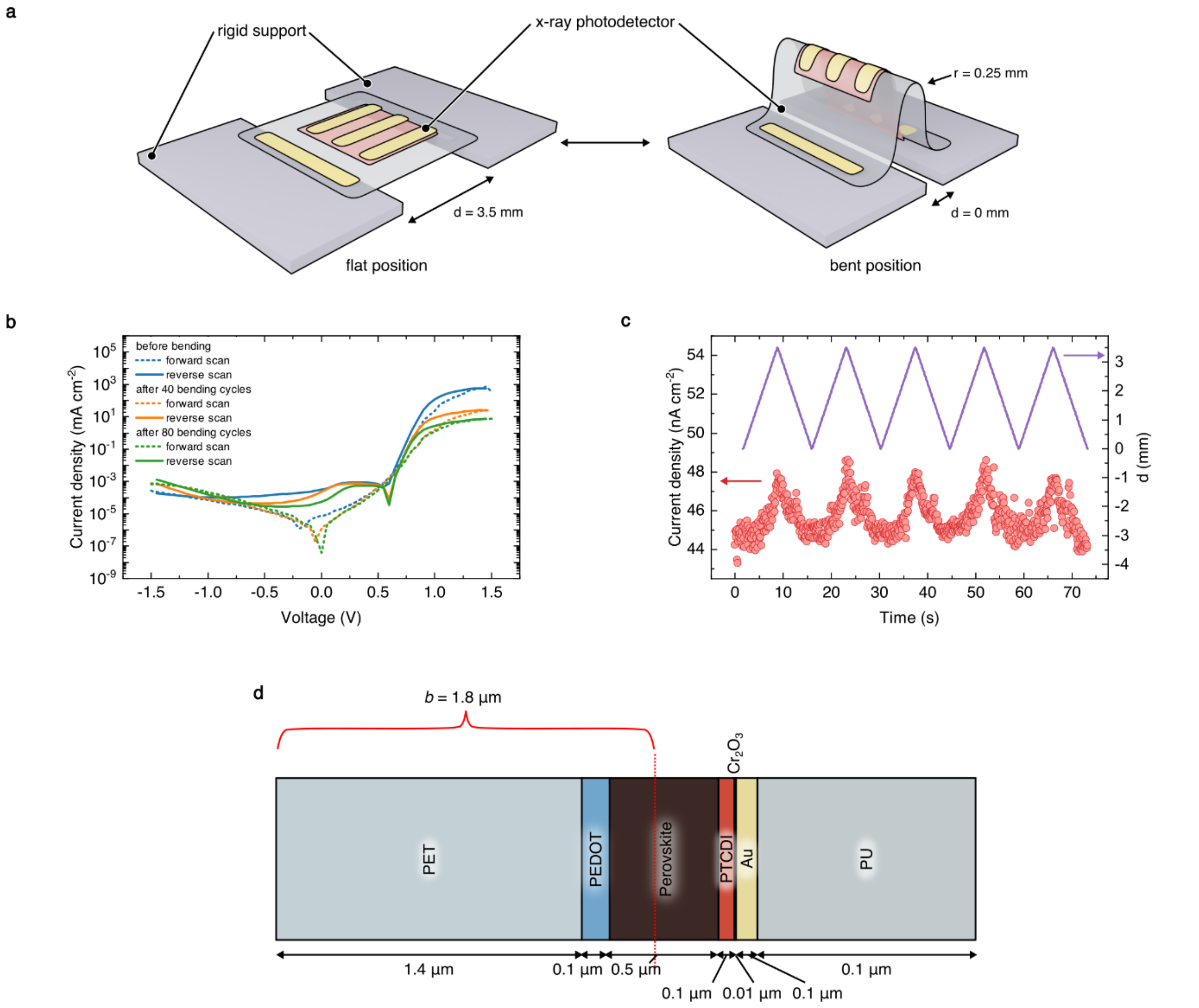

Figure S10: Assessment of bending flexibility performance of PTCDI/Cr_2O_3/Au type device. **a,** Schematic description of the bending set-up. The ultraflexible x-ray photodiode was mounted on rigid support plates and repeatedly bent by moving the plates with a help of linear actuator set-up. This enables the formation of a controlled wrinkle in the middle of ultraflexible x-ray photodiode with a bending radius as small as 0.25 mm. **b,** *JV* curve recorded before (blue) the bending experiment, after 40 (orange), and 80 (green) bending cycles. Minimal changes in the *JV* curve are observed at operating bias (-1÷0 V), even after repeated bending cycles can be attributed to positioning of the device in the neutral mechanical plane of the overall architecture, therefore protecting it from extreme mechanical stresses. **c,** Dynamic measurement of the dark current density (red) at -0.1 V applied bias as the device is bent 5 times (d - distance between rigid support plates, corresponding to bending cycles). **d,** X-ray photodiode architecture drawn to scale. The red dotted line marks the

location of neutral mechanical plane at 1.8 μm, that can be calculated using the following equation:

$$b = \frac{\sum_{i=1}^{n} \bar{E}_i h_i \left[(\sum_{j=1}^{i} h_j) - \frac{h_i}{2} \right]}{\sum_{i=1}^{n} \bar{E}_i h_i}$$

where 1st layer with n^{th} layer being on top, b –neutral mechanical plane as distance from the bottom PET substrate surface, $\bar{E} = \frac{E}{1-\upsilon^2}$ - plain strain module, E – Young's modulus, υ – Poisson's ratio, and h - layer thickness. Ultraflexible X-ray photodiode consists of 7 layers ($n = 7$) with corresponding thicknesses, Young's moduli and Poisson's ratios: $h_{PET} = 1.4\ \mu m$, $E_{PET} = 2.95\ GPa$, $\upsilon_{PET} = 0.34$, $h_{PEDOT} = 0.13\ \mu m$, $E_{PEDOT} = 2.60\ GPa$, $\upsilon_{PEDOT} = 0.33$, $h_{Perovskite} = 0.5\ \mu m$, $E_{Perovskite} = 20\ GPa$, $\upsilon_{Perovskite} = 0.39$, $h_{PTCDI} = 0.1\ \mu m$, $E_{PTCDI} = 2.30\ GPa$, $\upsilon_{PTCDI} = 0.31$, $h_{Cr} = 0.01\ \mu m$, $E_{Cr} = 294\ GPa$, $\upsilon_{Cr} = 0.20$, $h_{Au} = 0.1\ \mu m$, $E_{Au} = 79\ GPa$, $\upsilon_{Au} = 0.42$, $h_{PU} = 1\ \mu m$, $E_{PU} = 0.40\ GPa$, $\upsilon_{PU} = 0.39$.

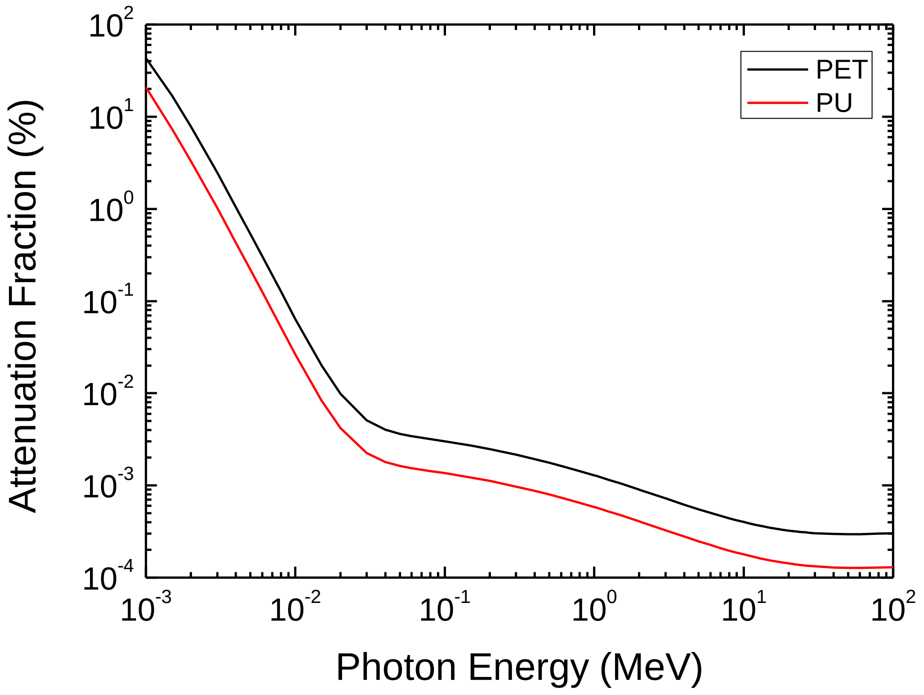

Figure S11: Attenuation fraction of 1.4 μm PET (black) and 1 PU (red) layers highlighting the reason for equivalent X-ray response from the front and back side of free standing ultrathin X-ray photodiodes.

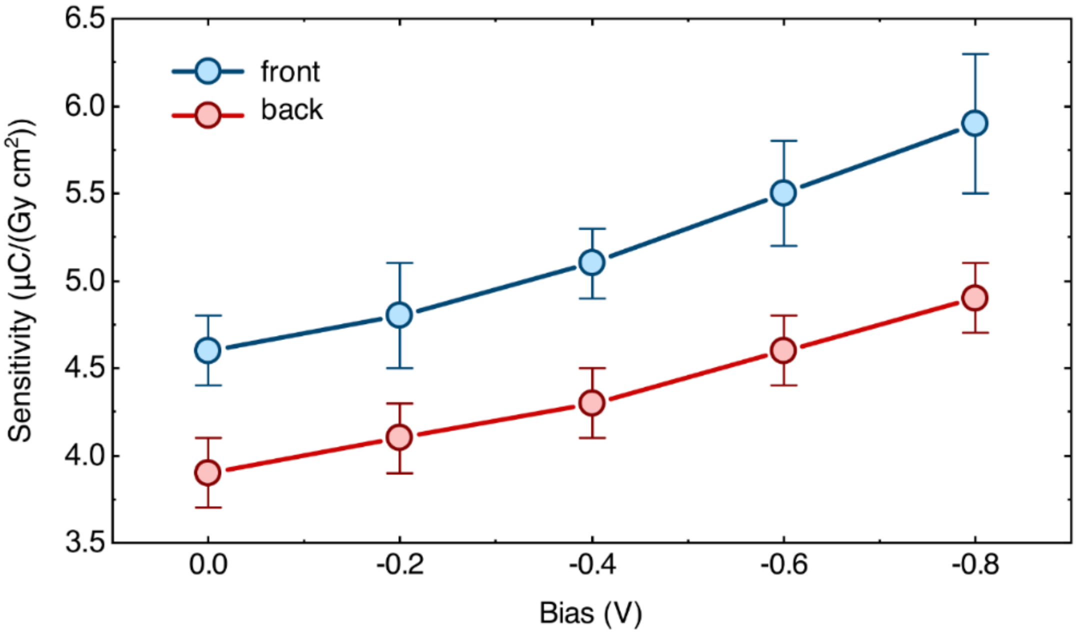

Figure S12: Plot of sensitivity as a function of bias for free-standing detectors in front (blue) and back (red) configurations.
